# CONSISTENT ASSIGNMENT: AN UPDATE ON THE QUALITY IMPACT

**DOI:** 10.1093/geroni/igz038.2950

**Published:** 2019-11-08

**Authors:** Nicholas Castle, John A Harris

**Affiliations:** 1 WVU, Morgantown, United States; 2 University of Pittsburgh, Pittsburgh, Pennsylvania, United States

## Abstract

The association of consistent assignment of Nurse Aides (NAs) with nursing home quality indicators is examined. Consistent assignment is defined as the same caregivers consistently caring for the same residents almost (80% of their shifts) every time they are on duty. Data used came from a survey of nursing home administrators, Nursing Home Compare, the Certification and Survey Provider Enhanced Reporting (CASPER) data, and the Area Resource File. All of the data was from 2015, and included 3,550 facilities. Several multivariate logistic regression models (using GEE) were used, including staffing variables (turnover, agency use, staffing levels), facility factors (size, ownership, occupancy rate), and market characteristics (competition, Medicaid rates). An average of 77% of nursing homes reported using at least some level of consistent assignment; although some were at low levels. In the multivariate analyses, accepted levels of consistent assignment were used. Turnover and family satisfaction quality were significantly (p<.01) better in facilities with the highest levels of consistent NA assignment. 7 of the 9 Quality Measures and 3 of the 5 Five-Star measures examined were significantly (p<.01) better in facilities with the highest levels of consistent NA assignment. Consistent assignment has developed as a preferred practice in nursing homes based on little empirical evidence. The findings presented here provide substantial justification for the use of this staffing practice for NAs.

